# Comparison of the gut microbiota and untargeted gut tissue metabolome of Chinese mitten crabs (*Eriocheir sinensis*) with different shell colors

**DOI:** 10.3389/fmicb.2023.1218152

**Published:** 2023-07-13

**Authors:** Xiaochen Zhu, Yingying Zhao, Na Sun, Changlei Li, Qing Jiang, Yazhao Zhang, Hua Wei, Yingdong Li, Qingbiao Hu, Xiaodong Li

**Affiliations:** ^1^College of Science and Engineering, Flinders University, Adelaide, SA, Australia; ^2^College of Animal Science and Veterinary Medicine, Shenyang Agricultural University, Shenyang, China; ^3^Liaoning Panjin Wetland Ecosystem National Observation and Research Station, Shenyang, China; ^4^Panjin Guanghe Crab Industry Co. Ltd., Panjin, China

**Keywords:** Chinese mitten crab, *Eriocheir sinensis*, gut microbiota, gut tissue metabolome, shell coloration

## Abstract

**Introduction:**

The Chinese mitten crab (*Eriocheir sinensis*) is a highly valued freshwater crustacean in China. While the natural shell color of *E. sinensis* is greenish brown (GH), we found a variety with a brownish-orange shell color (RH). Although RH is more expensive, it exhibits a lower molting frequency and growth rate compared with GH, which significantly reduces its yield and hinders large-scale farming. The growth and development of animals are closely related to their gut microbiota and gut tissue metabolic profiles.

**Methods:**

In this study, we compared the gut microbiome communities and metabolic profiles of juvenile RH and GH crabs using *16S rRNA* gene sequencing and liquid chromatography–mass spectrometry (LC–MS), respectively.

**Results:**

Our findings indicated that the intestinal microbial composition and metabolic characteristics of *E. sinensis* differed significantly between RH and GH. At the operational taxonomic unit (OTU) level, the α-diversity of the gut microbiota did not differ significantly between RH and GH, while the β-diversity of the RH gut microbiota was higher than that of the GH gut microbiota. At the species level, the richness of *unclassified_c_Alphaproteobacteria* was significantly higher in the GH group, while the RH group had a significantly higher richness of three low-abundance species, *Flavobacteria bacterium BAL38*, *Paraburkholderia ferrariae*, and *uncultured_bacterium_g__Legionella*. In the current study, 598 gut tissue metabolites were identified, and 159 metabolites were significantly different between GH and RH. The metabolite profile of RH was characteristic of a low level of most amino acids and lipid metabolites and a high level of several pigments compared with that of GH. These metabolites were enriched in 102 KEGG pathways. Four pathways, including (1) Central carbon metabolism in cancer, (2) protein digestion and absorption, (3) alanine, aspartate and glutamate metabolism, and (4) aminoacyl-tRNA biosynthesis, were significantly enriched. The correlation analysis between metabolites and microbiotas indicated that most key differential metabolites were positively correlated with the abundance of *Shewanella*_sp_MR-7.

**Discussion:**

This research provided a greater understanding of the physiological conditions of *E. sinensis* varieties with different shell colors by comparing the gut microbiota and gut tissue metabolome.

## 1. Introduction

The Chinese mitten crab (*Eriocheir sinensis*) is a highly sought-after freshwater crustacean in China due to its reputation as a delicacy (Wang et al., 2018). In 2019, the production of *E. sinensis* reached nearly 800,000 tons, with a market value of almost one billion US dollars (FAO, 2021). However, artificially selected variants are becoming increasingly important in industry due to the decline in wild populations (Wang et al., 2018). During a two-decade genetic improvement project, we found a variety with a brownish-orange shell color (RH), while the natural shell color of *E. sinensis* is greenish brown (GH). Because the brighter color of RH will undoubtedly increase its market price, RH has attracted the interest of aquarists, aquaculturists, and genetic breeders. Nevertheless, RH usually exhibits a lower molting frequency and growth rate than GH (Si et al., 2017), which significantly reduces its yield and hinders large-scale farming. Therefore, a deeper understanding of the genetic and physiological differences between RH and GH is necessary to develop a strategy to improve the performance of RH.

Omics technologies, such as transcriptomic, metabolomic, and gut microbiome analyses, provide innovative methodologies for investigating biological systems by probing and analyzing large datasets characterizing an organism under a particular condition (Dai and Shen, 2022; Jiang et al., 2022). In a previous study, we compared the transcriptome of first-stage zoea larvae (ZI) between RH and GH and identified differentially expressed genes (DEGs) that are associated with shell color differentiation and immunodeficiency (Zhao et al., 2020b). However, no further investigations have been conducted into other biological features or subsequent developmental stages.

The gut microbiota plays an essential role in organismal development, growth, homeostasis, and pathology because it significantly affects nutrition absorption, immune response, and organ development in the host (Sommer and Bäckhed, 2013; Liu et al., 2019). Gut microbiota composition is shaped by the host’s genetic background, developmental stage, health status, and environment (Ussar et al., 2016; Cahana and Iraqi, 2020; Li et al., 2021; Qin et al., 2022). Researchers have performed many studies comparing the gut microbiota of *E. sinensis* among different environments, diets, and ages and that of healthy and diseased individuals (Ding et al., 2017; Wang et al., 2019; Chen et al., 2021b; Shao et al., 2022; Xu et al., 2022). However, reports related to host genetic backgrounds are scarce. On the other hand, the gut microbiota contributes to host metabolic alteration and further affects host growth and development (Shi et al., 2019). The metabolite profile can not only reflect the physiological condition of the organism (Bundy et al., 2009; Wishart, 2019) but also define the functional status of the host and microbiota (Chen et al., 2019). Therefore, many studies have focused on *E. sinensis* metabolome variations related to food, disease, and the environment (Chen et al., 2021b; Guo et al., 2022; Jiang et al., 2022). In aquatic animals, integrative analysis of the microbiota and metabolome has been utilized to investigate alterations in the physiological status of hosts related to differences in nutrition intake (Chen et al., 2021b), habitats (Chen et al., 2021a), growth performance (Uengwetwanit et al., 2020; Lan et al., 2023), and aquaculture systems (Ding et al., 2022; Yokoyama et al., 2022).To our knowledge, there has been no metabolomic study related to the different shell colors of *E. sinensis* thus far. In this study, we investigated the gut microbiota and metabolomes of the gut tissue of juvenile RH and GH crabs. This comprehensive comparative analysis will enhance the understanding of the physiological state of RH and may offer a strategy to improve its performance.

## 2. Materials and methods

### 2.1. Sample collection

Healthy juvenile GH and RH *E. sinensis* crabs (9.5 ± 0.5 g) were obtained from Panjin Guanghe Crab Industry Co., Ltd., in Panjin, China, in April 2021. Prior to collection, these crabs were raised in the indoor tanks (8,000 L) at a density of 60 crabs per m^3^ under the following condition, dissolved oxygen >5.0 mg/ L, pH 8.3–8.8, and ammonia < 0.2 mg/L for 6 months. The crabs were fed twice daily with commercial pelleted feed (Wellhope Aquatic Feed Co. Ltd., Shenyang, China). Upon arrival at the lab, they were temporally raised in the same tank under continuous aeration at approximately 21°C. To clear the gut contents and normalize the metabolic status, the crabs were fasted for 24 h. Twelve individuals were randomly selected from each color group. We washed each crab’s body surface with sterile water and dissected it immediately. Approximately 1.5 cm of mid- and hindgut was collected with sterile instruments. Two individual gut samples were pooled as a single sample and placed in a 2 mL cryogenic vial, submerged in liquid nitrogen, and stored at −80°C for gut microbiota sequencing and liquid chromatography–mass spectrometry (LC–MS)-based metabolomic analysis.

### 2.2. Gut microbiota sequencing and data processing

Microbial community genomic DNA was extracted from 12 *E. sinensis* gut samples (six from each color) with an E.Z.N.A.® soil DNA Kit (Omega Biotek, Norcross, GA, USA) following the manufacturer’s instructions. DNA concentration and purity were measured by a NanoDrop 2000 (Thermo Scientific, Wilmington, USA). The hypervariable region V3-V4 of the bacterial *16S rRNA* gene was amplified with the primer pair 338F (5’-ACTCCTACGGGAGGC AGCAG-3′) and 806R (5′-GGACTACHVGGGTWTCTAAT-3′) with the same PCR program as described in a previous study (Chen et al., 2021b). The PCR product was purified using the AxyPrep DNA Gel Extraction Kit (Axygen Biosciences, Union City, CA, USA) according to the manufacturer’s instructions and quantified using a Quantus™ Fluorometer (Promega, USA).

Purified amplicons were pooled in equimolar amounts and paired-end sequenced on an Illumina MiSeq PE300 platform (Illumina, San Diego, USA) with standard protocols by Majorbio Bio-Pharm Technology Co., Ltd. (Shanghai, China). The raw reads were deposited into the NCBI Sequence Read Archive (SRA) database (accession numbers: PRJNA957701 and PRJNA970672).

After sequencing, the raw *16S rRNA* gene sequencing reads were demultiplexed and quality-filtered by fastp version 0.20.0 (Chen et al., 2018). The reads were merged by FLASH (1.2.11; Magoc and Salzberg, 2011) with the same criteria as described in a previous study (Zhu et al., 2022). Operational taxonomic units (OTUs) were clustered using UPARSE version 7.1 (Edgar, 2013) with a 97% similarity cutoff, and chimeric sequences were identified and removed. The taxonomy of each representative OTU sequence was analyzed by RDP Classifier version 2.2 (Wang et al., 2007) against the *16S rRNA* database (Silva SSU 138) using a confidence threshold of 0.7.

The Chao and Shannon indices for α-diversity were calculated by Mothur 1.30.2 (Schloss et al., 2009). The β-diversity was determined through Bray–Curtis dissimilarity matrices and visualized using principal coordinate analysis (PCoA). To identify differences between groups, we conducted an analysis of similarities (ANOSIM) using Bray–Curtis dissimilarity. Functional prediction for gut bacteria was performed using PICRUSt2 (Douglas et al., 2020). The differential functions between RH and GH were measured through Student’s t test of relative abundances. All of these analyses were carried out on the Majorbio I-Sanger Cloud Platform (Ren et al., 2022).

### 2.3. LC–MS metabolomic processing and data analysis

In this study, the residual gut tissue from 12 samples was used for LC–MS metabolomic processing. To extract the metabolites, 50 mg of gut tissue was mixed with a 400 mL solution of methanol:water (4:1, v/v) containing 0.02 mg/mL L-2-chlorophenylalanin as the internal standard. The mixture was then subjected to 6 min of crushing at 50 Hz using a Wonbio-96c high-throughput tissue crusher (Shanghai Wanbo Biotechnology Co. Ltd), followed by ultrasonication at 40 kHz for 30 min at 5°C. The samples were then placed at −20°C for 30 min to precipitate proteins. After centrifugation, the supernatant was transferred to sample vials and analyzed using the UHPLC-Q Exactive system from Thermo Fisher Scientific. Chromatography was carried out according to the standard protocol of Majorbio Bio-Pharm Technology Co., Ltd. (Shanghai, China).

Following mass spectrometric detection, the raw LC/MS data were preprocessed by Progenesis QI (Waters Corporation, Milford, USA). The data were searched and identified in the following databases: human metabolome database (HMDB)^1^, Metlin^2^, and the Majorbio Database. The resulting data were uploaded to the Majorbio cloud platform^3^ (Ren et al., 2022) for further analysis. First, data were filtered by retaining those metabolites detected in at least 80% of any sample set. Missing data were replaced by the minimum value, and metabolic features were normalized by sum. The response intensity of sample mass spectrum peaks was normalized by the sum normalization method, and variables with relative standard deviation (RSD) > 30% of the quality control (QC) were removed. Finally, log10 transformation was applied to generate the final data matrix.

The least partial squares discriminant analysis (PLS-DA) was carried out using the R package ropls (Version 1.6.2). The significant differences in the metabolites were determined based on the variable importance in the projection (VIP) score obtained from the PLS-DA model and the *p* value from Student’s t test. Metabolites with a VIP score greater than 1 and a value of *p* less than 0.05 were considered significantly different.

Differential metabolites between RH and GH were categorized in the Kyoto Encyclopedia of Genes and Genomes (KEGG) database^4^ and HMDB.^5^ Differential metabolites were mapped to biochemical pathways through metabolic enrichment and pathway analysis based on the KEGG database.^6^ Then, scipy.stats (Python packages)^7^ was used to identify statistically significantly enriched pathways using Fisher’s exact test. The above procedures were performed using the Majorbio I-Sanger Cloud Platform (Ren et al., 2022).

### 2.4. Correlation analysis between the gut microbiota and metabolite profile

The differential metabolites in the significantly enriched pathways were considered key differential metabolites. Spearman correlation coefficients between gut microbes (at the species level) and 11 key differential metabolites as well as five pigments and dopaquinone (melanogenesis related) were calculated and visualized by the corrplot package in R (1.6.2).

## 3. Results

### 3.1. Comparison of gut microbiota diversity between crabs with different shell colors

**Figure 1:**
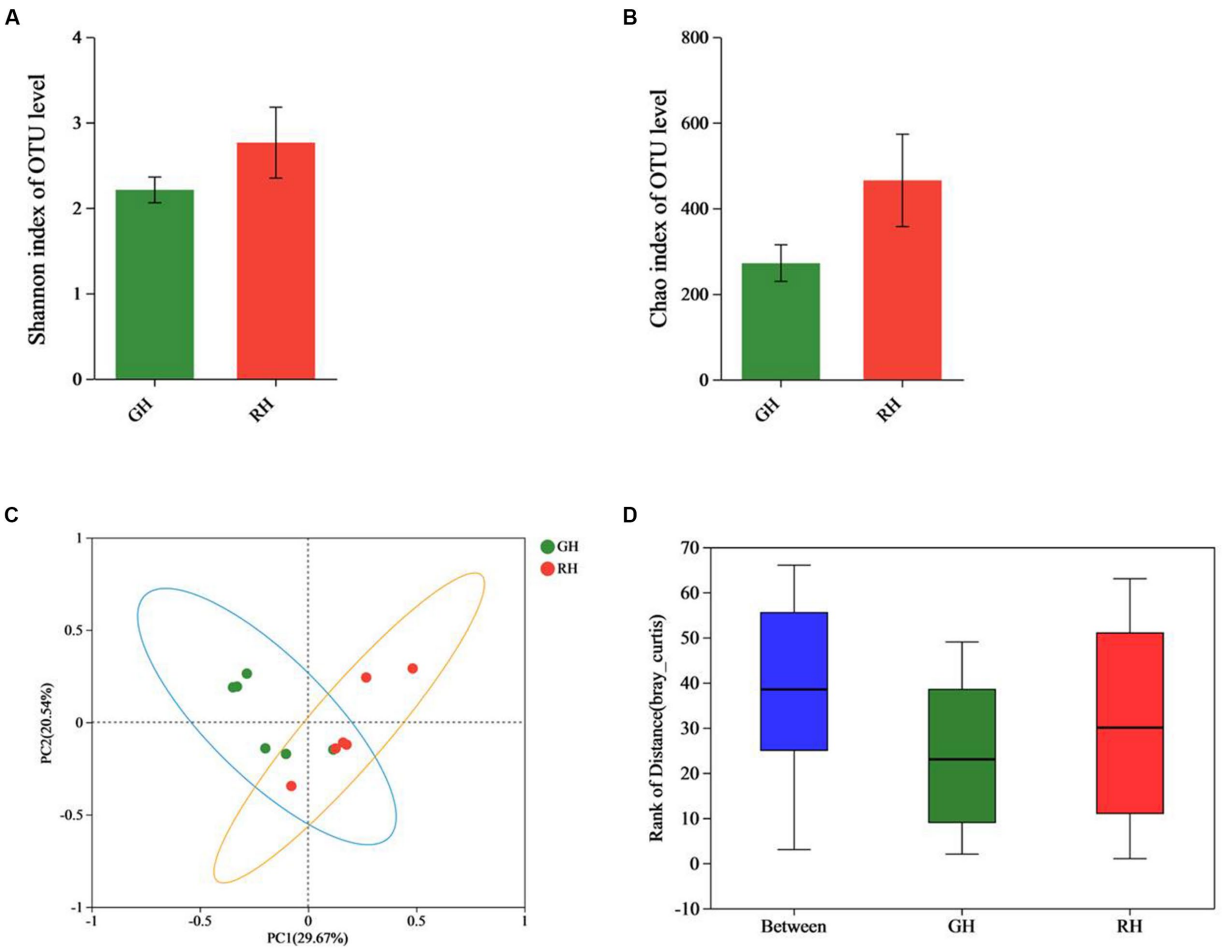
Comparison of gut microbiota diversity between GH and RH at the OTU level. **(A)** α-Diversity (Shannon index) estimate. **(B)** α-Diversity (Chao index) estimate. **(C)** β-Diversity estimate (PCoA). **(D)** β-Diversity estimate (ANOSIM).

After sequencing each sample, an average of 46,390 raw reads were generated, resulting in a total of 556,675 raw reads. After filtering, 33,516 OTUs were identified. The Shannon rarefaction curve between the number of reads and the Shannon index at the OTU level attained a saturation Plateau for each sample (Supplementary Figure 1). The Shannon and Chao indices showed no significant difference in α-diversity between RH and GH at the OTU level (*p* > 0.05; Figures 1A,B). ANOSIM analysis and β-diversity analysis through PCoA indicated significant differences between the two groups (*R* = 0.3481, *p* = 0.012; Figures 1C,D).

### 3.2. Bacterial community compositions in crabs with different shell colors

**Figure 2:**
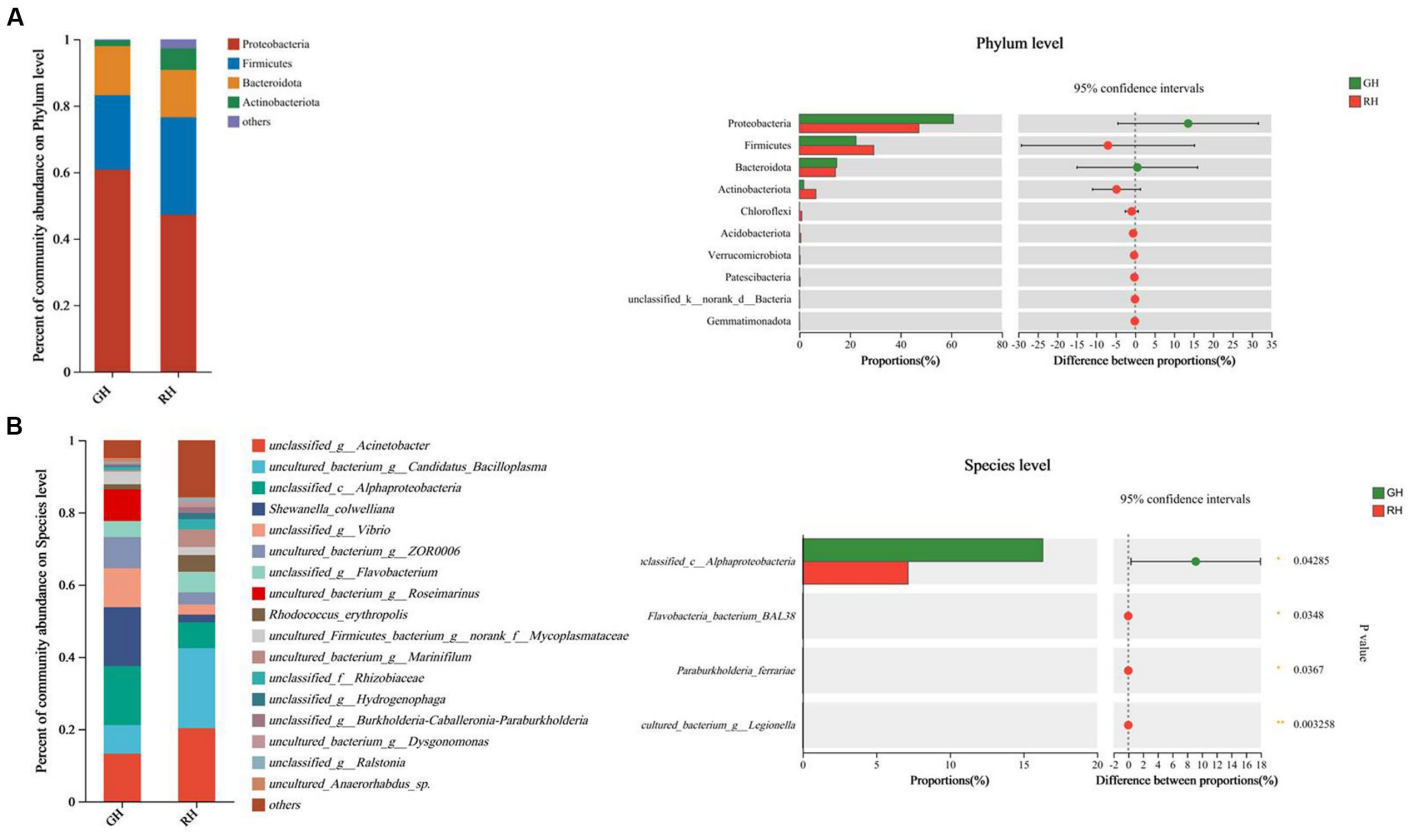
Gut microbiota composition and richness of GH and RH. **(A)** Gut microbiota composition and richness at the phylum level (top 10 in richness). **(B)** Gut microbiota composition and richness at the species level (significantly different between RH and GH).

In both the GH and RH groups, Proteobacteria, Firmicutes and Bacteroidota were found to be the most dominant phyla, as indicated in Figure 2A. The abundance of Proteobacteria, Firmicutes, and Bacteroidota in the GH group was 60.82, 22.37, and 14.76%, respectively, while that in the RH group was 47.21, 29.36, and 14.20%, respectively. However, no significant differences between the GH and RH groups were observed in the gut microbiome communities (Figure 2A).

At the species level, *Shewanella_colwelliana* (16.32%), *unclassified_c__Alphaproteobacteria* (16.31%), and *unclassified_g__Acinetobacter* (13.22%) were the three predominant species in the GH group (Figure 2B). The three predominant species in the RH group were *uncultured_bacterium_g__Candidatus_Bacilloplasma* (22.16%), *unclassified_g__Acinetobacter* (20.26%), and *unclassified_c_Alphaproteobacteria* (7.15%; Figure 2B). Among the identified species, the richness of *unclassified_c_Alphaproteobacteria* was significantly higher in the GH group, while the RH group had a significantly higher richness of three low-abundance species, namely, *Flavobacteria bacterium BAL38, Paraburkholderia ferrariae,* and *uncultured_bacterium_g_Legionella* (*p* < 0.05; Figure 2B).

Based on their *16S rRNA* sequences, the gut microbiota functional profiles of RH and GH were predicted. As a result, a significant difference in gut microbiota functional abundance was identified between GH and RH in two pathways, namely, amino acid metabolism and drug resistance (*p* < 0.05, Supplementary Figure 2).

### 3.3. Comparison of gut tissue metabolomes in crabs with different shell colors

**Figure 3:**
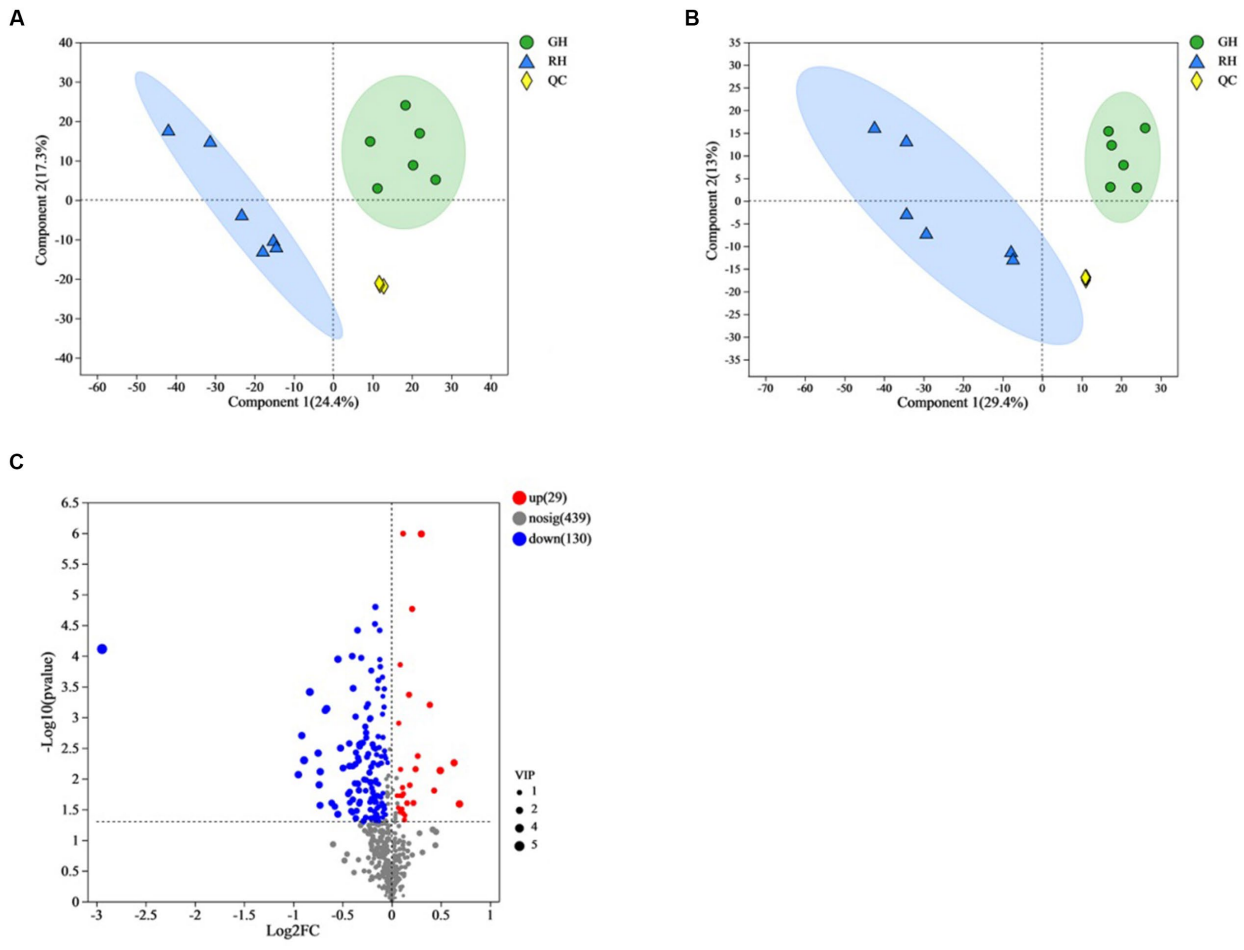
Overview and comparison of the gut tissue metabolomic profiles in the two shell color groups. **(A)** Score plots of PLS-DA in positive mode using identified metabolite data. **(B)** Score plots of PLS-DA in negative mode using identified metabolite data. **(C)** Heatmap cluster analysis for identified differential metabolites. QC, quality control.

In this study, a total of 6,135 metabolites were identified in positive ionization mode, and 5,783 metabolites were identified in negative ionization mode. After annotation, 598 metabolites were found to be known metabolites, out of which 315 were identified in positive-ionization mode and 285 were identified in negative-ionization mode. PLS-DA score plots were created to display the differences between the two groups. The positive and negative ionization mode data showed a clear distinction and discrimination between the two groups (Figures 3A,B).

Out of the 598 identified metabolites, 159 metabolites (67 in positive ionization mode and 92 in negative ionization mode) were significantly different between GH and RH (Supplementary Table 1). In RH, 29 metabolites were upregulated while 130 were downregulated in comparison to GH (Figure 3C).

**Figure 4:**
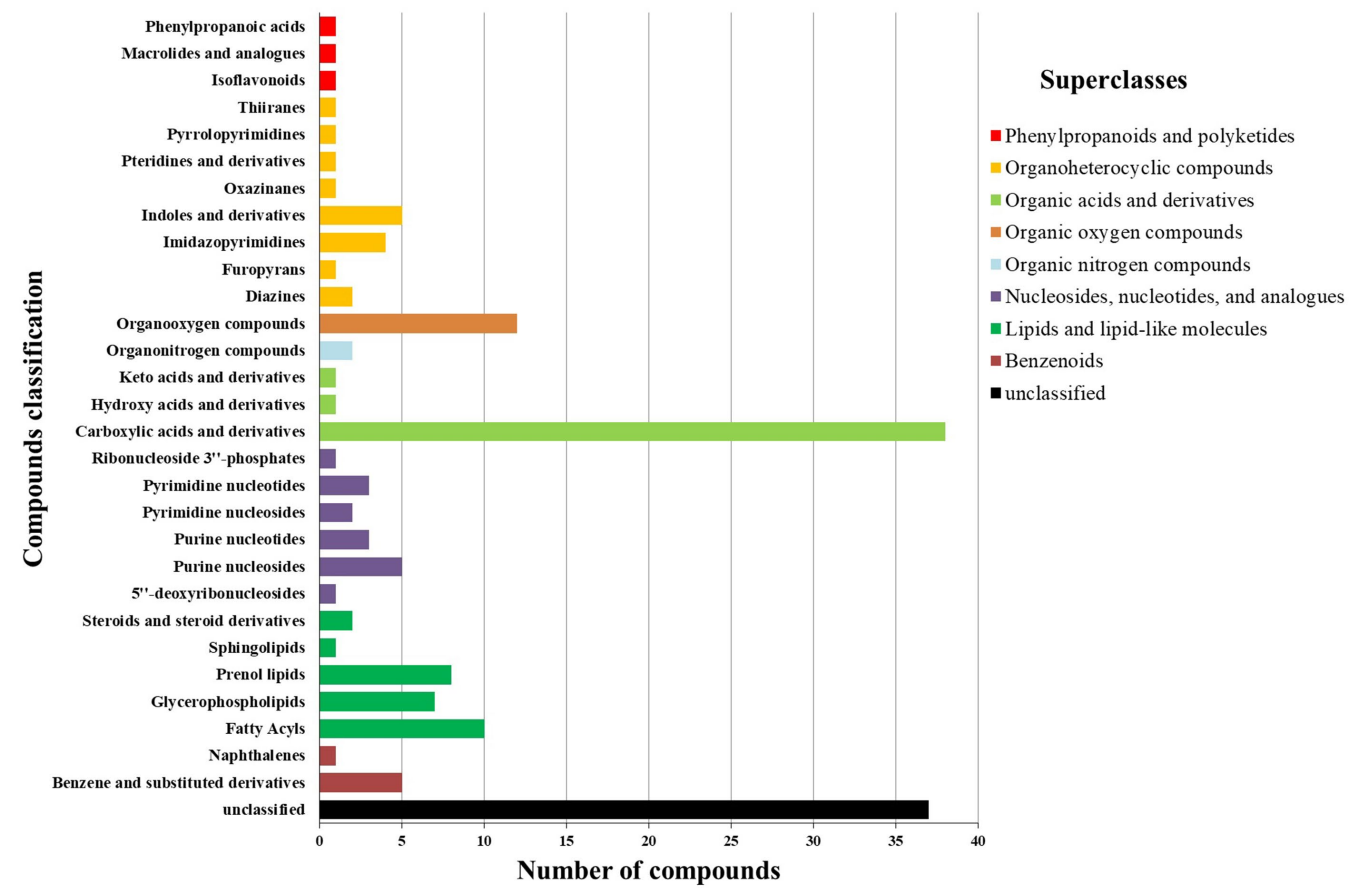
HMDB compound classification of differential metabolites between RH and GH.

**Figure 5:**
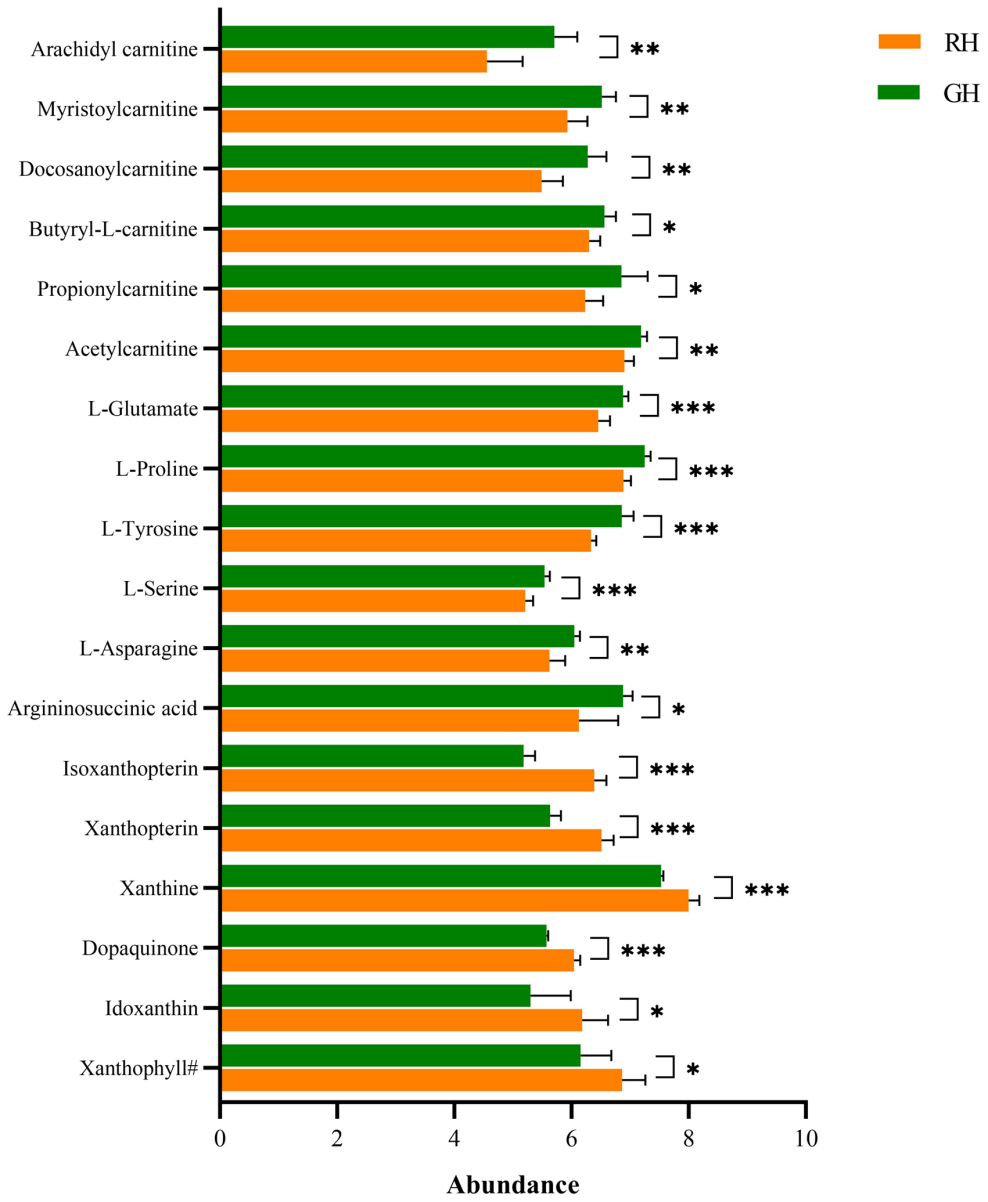
Differential metabolites between RH and GH. *p < 0.05; **p < 0.01; ***p < 0.001. Xanthophyll#, (3S,3’S,5R,5’R,6R)-3,6-Epoxy-5,6-dihydro-3′,5,8′-trihydroxy-beta,kappa-caroten-6′-one.

Except for unclassifiable metabolites, the differential metabolites were classified into eight HMDB superclasses (Figure 4). Most differential metabolites belonged to the classes of organic acids and derivatives (40) and lipids and lipid-like molecules (28). Among the 28 lipids and lipid-like molecules, only three were upregulated in RH, including (3S,3’S,5R,5’R,6R)-3,6-epoxy-5,6-dihydro-3′,5,8′-trihydroxy-beta,kappa-caroten-6′-one (xanthophyll^#^), idoxanthin (Figure 5) and LysoPC (22:4 (7Z,10Z,13Z,16Z)). Notably, low levels of acylcarnitines, including acetylcarnitine, propionylcarnitine, butyrylcarnitine, docosanoylcarnitine, myristoylcarnitine, and arachidyl carnitine, were observed in the RH group (Figure 5). In the class of organic acids and derivatives, only four out of 40 were upregulated, including dopaquinone (Figure 5).

**Figure 6:**
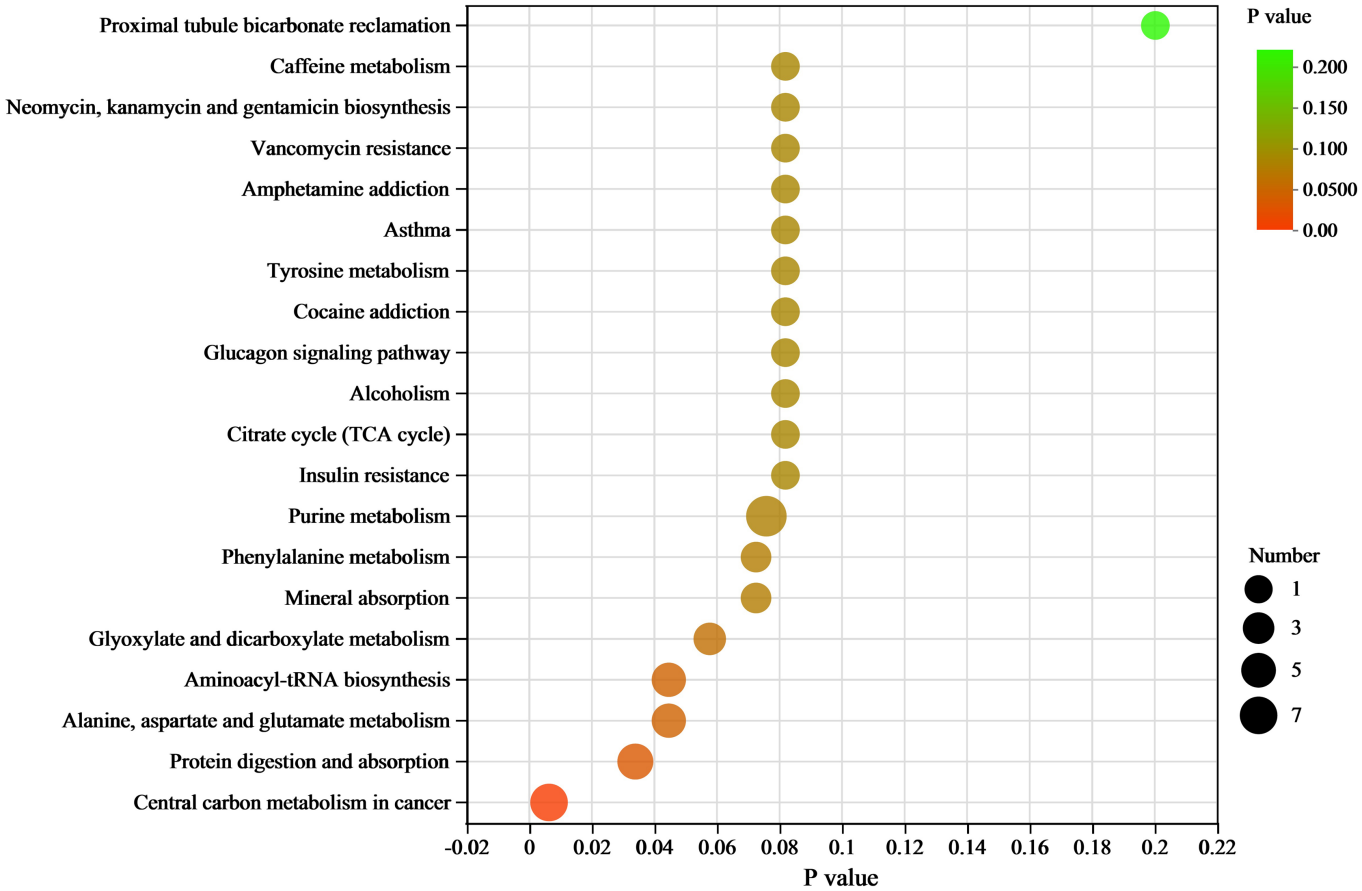
KEGG pathway enrichment scatter plot of differential metabolites between RH vs. GH. The scatter size indicates the metabolite number.

In the KEGG pathway analysis, differential metabolites were enriched in 102 KEGG pathways (Supplementary Table 2). The top 20 pathways and their differential metabolite numbers are shown in Figure 6. The greatest number of enriched metabolites (9) was observed in purine metabolism. However, only four pathways, including (1) Central carbon metabolism in cancer, (2) protein digestion and absorption, (3) alanine, aspartate and glutamate metabolism, and (4) aminoacyl-tRNA biosynthesis, were significantly enriched (*p* < 0.05). Nine of the 11 key differential metabolites belonged to the subclass of amino acids, peptides, and analogues.

### 3.4. Correlation analysis between the gut microbiome and metabolite profiles

**Figure 7:**
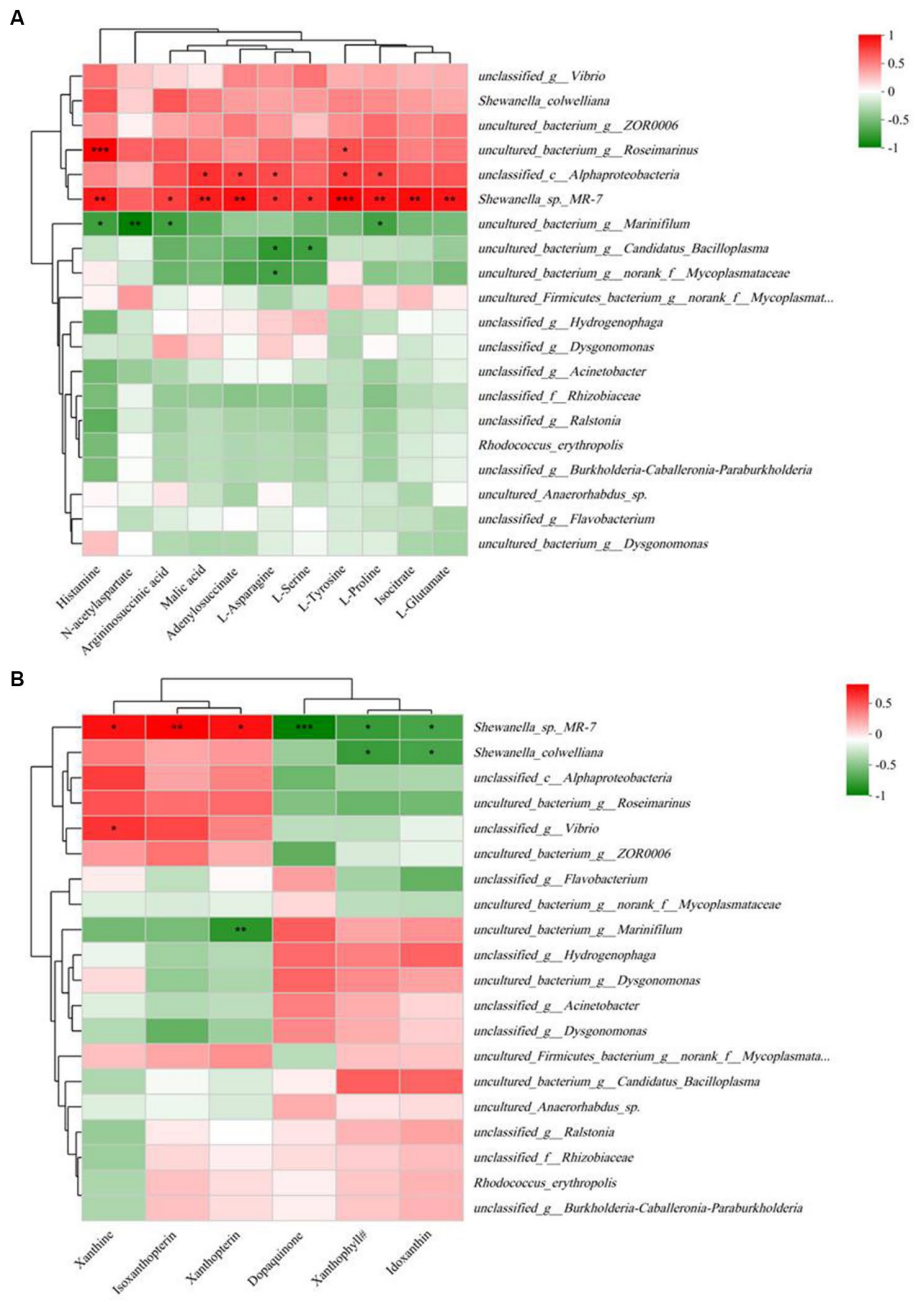
Spearman correlation heatmap based on the differential bacterial community at the species level and 11 key differential metabolites in significantly different KEGG pathways **(A)** and five pigments and dopaquinone **(B)**. The color scale on the right shows the color partitioning of the different *R* values. *p < 0.05; **p < 0.01; ***p < 0.001. Xanthophyll^#^, (3S,3’S,5R,5’R,6R)-3,6-Epoxy-5,6-dihydro-3′,5,8′-trihydroxy-beta,kappa-caroten-6′-one.

Correlation analysis between 11 key differential metabolites and gut microbiota profiles (at the species level) is shown in Figure 7A. The results indicated that all 11 key metabolites except N-acetylaspartate were significantly positively correlated with *Shewanella_sp*_MR-7. Histamine, N-acetylaspartate, arginosuccinic acid, and L-proline were significantly negatively correlated with *uncultured_bacterium_g_Marinifilum*, while histamine and L-tyrosine were significantly positively correlated with *uncultured_bacterium_g_Roseimarinus*.

The results of correlation analysis between the five pigments and dopaquinone and the gut microbiota profile (at the species level) are shown in Figure 7B. The results indicated that *Shewanella*_sp_MR-7 was positively correlated with xanthine, isoxanthopterin, and xanthopterin, but negatively correlated with dopaquinone, xanthophyll^#^, and idoxanthin.

## 4. Discussion

The gut microbiota is essential for host physiological functions, such as nutrition acquisition, development, homeostasis, and immunity (Butt and Volkoff, 2019). It can be influenced by several factors, including genetic background, sex, developmental stage, habitat environment, feed type, health conditions, and external stimuli (Piazzon et al., 2020). While nongenetic factors have been extensively studied for their impact on the changes in the intestinal microorganisms of *E. sinensis* (Chen et al., 2021a,b; Guan et al., 2021; Guo et al., 2021; Jiang et al., 2022; Shao et al., 2022), little research has been conducted on the effects of host genetic background on the composition of the intestinal microflora in this economically significant species. This study investigated the gut microbiome communities and metabolic profiles in juveniles of two *E. sinensis* crab varieties with different shell colors. Our findings indicated that the intestinal microbial composition and metabolic characteristics of *E. sinensis* differed significantly between RH and GH.

In the present study, the intestinal microbiome communities of *E. sinensis* with different shell colors living in the same environment were not significantly different at the phylum level. The predominant microbial taxa in both GH and RH were Proteobacteria, Firmicutes, and Bacteroidota, which is consistent with previous studies on the *E. sinensis* gut microbiota (Guo et al., 2021; Shao et al., 2022). The intestine is an essential organ for nutrition digestion and absorption, and gut microorganisms are a crucial component in dietary metabolism (Hsiao et al., 2008). Previous studies have indicated that Firmicutes and Bacteroidetes enhance lipid metabolism and carbohydrate digestion, respectively (Turnbaugh et al., 2006; Karlsson et al., 2011; Chen et al., 2021a,b). Different diets contribute to significantly different compositions of these two phyla (Chen et al., 2021b). In this study, Proteobacteria, Firmicutes and Bacteroidota were found to be the predominant phyla in both GH and RH, and the abundance of each phylum did not significantly differ between groups. The gut microbiota of *E. sinensis* is dominated by Proteobacteria, which has been observed in other aquatic animals, including fishes (Kim et al., 2021) and other crustaceans (Fan et al., 2019; Apine et al., 2021). In mammals, a bloom of Proteobacteria is a sign of instability or dysbiosis of the gut microbiota because many members of commensal Proteobacteria can become pathobionts (Shin et al., 2015). For aquatic animals, however, a high level of Proteobacteria is common and probably associated with their unsegmented digestive system (Sunagawa et al., 2015). In addition, the carnivorous diet habit of *E. sinensis* also leads to a high abundance of Proteobacteria (Gao et al., 2020; Chen et al., 2021a).

In this study, the metabolomic profile of *E. sinensis* was also related to its shell color type, showing clear separation and discrimination. A total of 159 differential metabolites were identified, most of which were organic acids and derivatives (40) and lipids and lipid-like molecules (28), except for those that were unidentifiable. Amino acids serve as the main building blocks of proteins and play a significant role in crustacean growth, development and innate immune response (Huang et al., 2020). In RH, 33 out of 37 amino acids, peptides, and analogs (e.g., L-proline, L-asparagine, L-tyrosine, L-serine, and L-glutamate) exhibited lower levels. The unique cyclic structure of proline makes it a crucial component for maintaining the stability of protein structures. Moreover, it plays a significant role in the synthesis of arginine, polyamines, and glutamate, which are important for cellular metabolism. Additionally, proline has been shown to have antioxidative properties and contributes to immune responses and wound healing processes (Wu et al., 2011; Huang et al., 2020). Tyrosine is not only involved in protein synthesis and antioxidant activity but also functions as a precursor for neurotransmitters and melanin synthesis (Slominski et al., 2012). Glutamate plays a critical role in protein structure, signaling, metabolism and nutrition (Brosnan and Brosnan, 2013; Zhao et al., 2019). In addition, these five amino acids are also involved in the TCA cycle (Sherry et al., 2015). The TCA cycle is the essential metabolic pathway that connects amino acid, fat and carbohydrate metabolism in aerobic organisms (Arnold and Finley, 2023). In this study, the decrease in amino acid levels and the other two TCA cycle intermediates (isocitrate and malate) in the RH group affected the TCA cycle and consequently the energy supply efficiency. This possibly contributed to the low growth rate of RH. Given the general reduction in amino acids in the RH group, dietary supplementation with specific amino acids might be a viable approach for improving the growth and immunity of RH. In previous studies, dietary glutamate supplementation improved the growth performance of many fish (Oehme et al., 2010; Yoshida et al., 2016; Zhao et al., 2020a). Proline supplementation enhanced the immune response and antioxidant capability of juvenile Pacific white shrimp (Xie et al., 2015). In addition, supplementation with other essential amino acids (i.e., arginine and lysine) also positively affected the weight gain (WG) and specific growth rate (SGR) of some crustaceans (Zhou et al., 2012; Jin et al., 2015, 2016).

Similar to amino acids, most of the lipid and lipid-like molecules were decreased in RH. Notably, low levels of six acylcarnitines were observed in RH. Acylcarnitines are fatty acid metabolites that play a crucial role in balancing intracellular sugar and lipid metabolism. By serving as carriers, they transport long-chain fatty acids into mitochondria for β-oxidation, which generates a significant amount of energy for various cell activities (Li et al., 2019; Dambrova et al., 2022). Considering that RH and GH were subjected to the same culture conditions and fed the same diet, it is possible that RH has a lower efficacy in nutrient utilization, which contributes to its slower growth rate. Accordingly, dietary supplementation with carnitine may be a suitable method to improve nutrient metabolism in RH. In a previous study, carnitine supplementation improved the growth performance and food utilization of narrow-clawed crayfish (Safari et al., 2015).

Correlation analysis between 11 key differential metabolites and gut microbiota profiles indicated that almost all of the key differential metabolites were significantly positively correlated with *Shewanella*_sp_MR-7. As a probiotic, *Shewanella*_sp_MR-7 has been supplemented in the Pacific white shrimp diet and shown to positively enhance their growth performance, intestinal microbiota, and immunity (Wei et al., 2021). The potential for altering metabolite levels by elevating the proportion of *Shewanella_sp*_MR-7 in the RH microbiota needs to be further verified experimentally.

In this study, we also observed a significant excess of yellowish pigments in RH compared with GH, which may be associate with its brownish-orange color. These pigments included two xanthophylls (xanthophyll^#^ and idoxanthin), xanthopterin, and xanthine. Xanthophylls are a class of oxygen-containing carotenoid pigments and display a yellowish color. In a previous study, Liu et al. (2020) suggested that a higher total carotenoid content in the gut tissue of a scallop variant presenting gold color was correlated with the abundance of carotenoid-producing bacteria of the genus *Brevundimonas*. We also identified several microbiota species that were correlated with these pigments (Figure 7B). However, whether these species produce those pigments needs further investigation. Dopaquinone plays a pivotal role in melanogenesis (Ito and Wakamatsu, 2008) because it is the precursor of both eumelanin (which appears black to brown) and pheomelanin (which appears yellow to reddish) (Simon and Peles, 2010; D’Alba and Shawkey, 2019). The production of pheomelanin requires tyrosinase and cysteine, while the production of eumelanin requires tyrosinase and tyrosinase-related proteins 1 and 2 (Ito and Wakamatsu, 2008). Similarly, the catabolism of yellow pigments, xanthine, and xanthopterin, requires xanthine oxidase and xanthine dehydrogenase (Watt, 1972; Chung et al., 1997). Thus, these genes are likely involved in the coloration of *E. sinensis.* Further investigation of the genotype and expression of these genes might help illustrate the genetic basis of the coloration of RH.

In this study, we compared the gut microbiota and gut tissue metabolites of juvenile *E. sinensis* between RH and GH. The gut microbiota analysis indicated that the difference in richness between RH and GH stemmed from *unclassified_c_Alphaproteobacteria* and three low-abundance species. Gut tissue metabolite profiles suggested a difference in nutrient utilization between RH and GH. In addition, the excess pigments (e.g., xanthophylls, xanthine) in RH suggested a possible difference (in either genotype or expression) in the genes associated with their metabolism. Further investigations on these genes may help illustrate the genetic basis of *E. sinensis* shell coloration. In summary, this study enhances the understanding of the physiological conditions of *E. sinensis* crabs with different shell colors and provides guidance for RH cultivation to improve its growth performance.

## Data availability statement

The data presented in the study are deposited in the NCBI Sequences Read Archive (SRA) repository, accession numbers PRJNA957701 and PRJNA970672.

## Ethics statement

The animal study was reviewed and approved by Institutional Animal Care and Use Committee (IACUC) of Shenyang Agricultural University. All efforts were made to minimize the suffering of the animals.

## Author contributions

YiZ conceived and designed the experiments. CL, QJ, and YaZ performed the experiments. NS, CL, QJ, and YaZ collected the samples. XZ and YiZ analyzed the data and wrote the manuscript. YiZ and XL provided reagents and materials. HW, YiL, and QH revised the manuscript. All authors read and approved the final manuscript.

## Funding

This research work was supported by grants from Overseas Training Program for Colleges and Universities of Liaoning Province (2020GJWYB017), Liaoning Province “The Open Competition Mechanism to Select the Best Candidates” Project (2021JH1/10400040), and the National Natural Science Foundation of China (No. 32002314).

## Conflict of interest

NS and XL were employed by Panjin Guanghe Crab Industry Co. Ltd.

The remaining authors declare that the research was conducted in the absence of any commercial or financial relationships that could be construed as a potential conflict of interest.

## Publisher’s note

All claims expressed in this article are solely those of the authors and do not necessarily represent those of their affiliated organizations, or those of the publisher, the editors and the reviewers. Any product that may be evaluated in this article, or claim that may be made by its manufacturer, is not guaranteed or endorsed by the publisher.

## Footnotes

^1^ http://www.hmdb.ca

^2^ http://metlin.scripps.edu

^3^ https://cloud.majorbio.com

^4^ http://www.genome.jp/kegg/

^5^ www.hmdb.ca

^6^ http://www.genome.jp/kegg/

^7^ https://docs.scipy.org/doc/scipy/
