# Supplementary figures and images for: Comparison of the gut microbiota and untargeted gut tissue metabolome of Chinese mitten crabs (Eriocheir sinensis) with different shell colors
Source: Front Microbiol. 2023 Jul 13;14:1218152. doi: 10.3389/fmicb.2023.1218152 (PMC10374289; doi:10.3389/fmicb.2023.1218152)

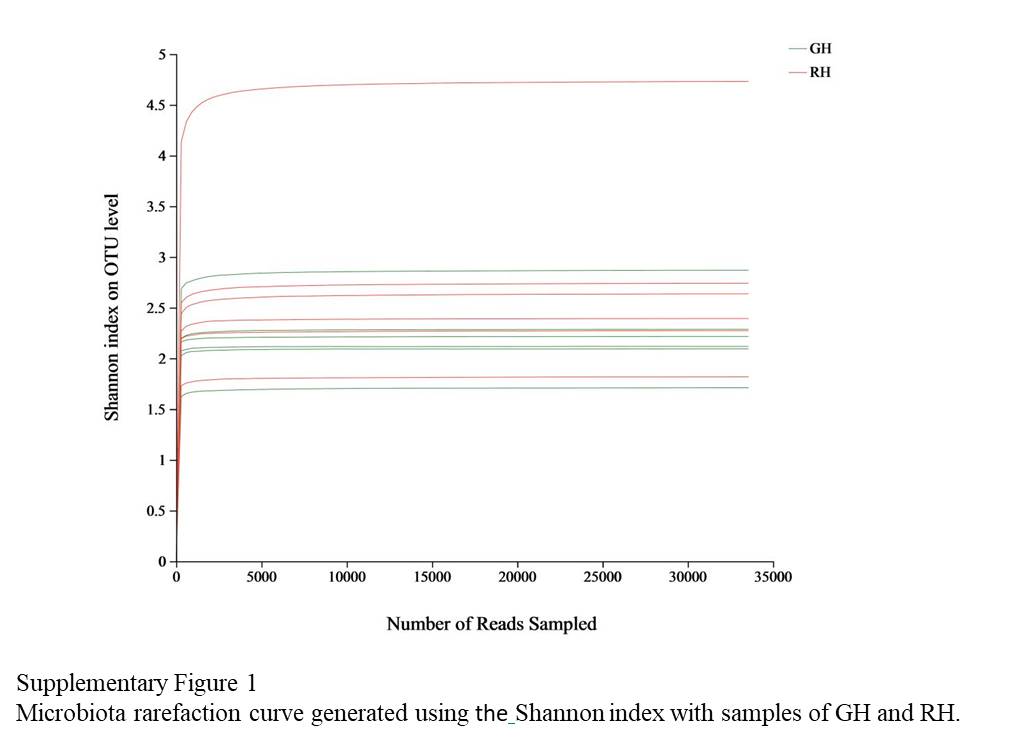

Supplement: Supplementary file 3 [file Image_1.jpg]

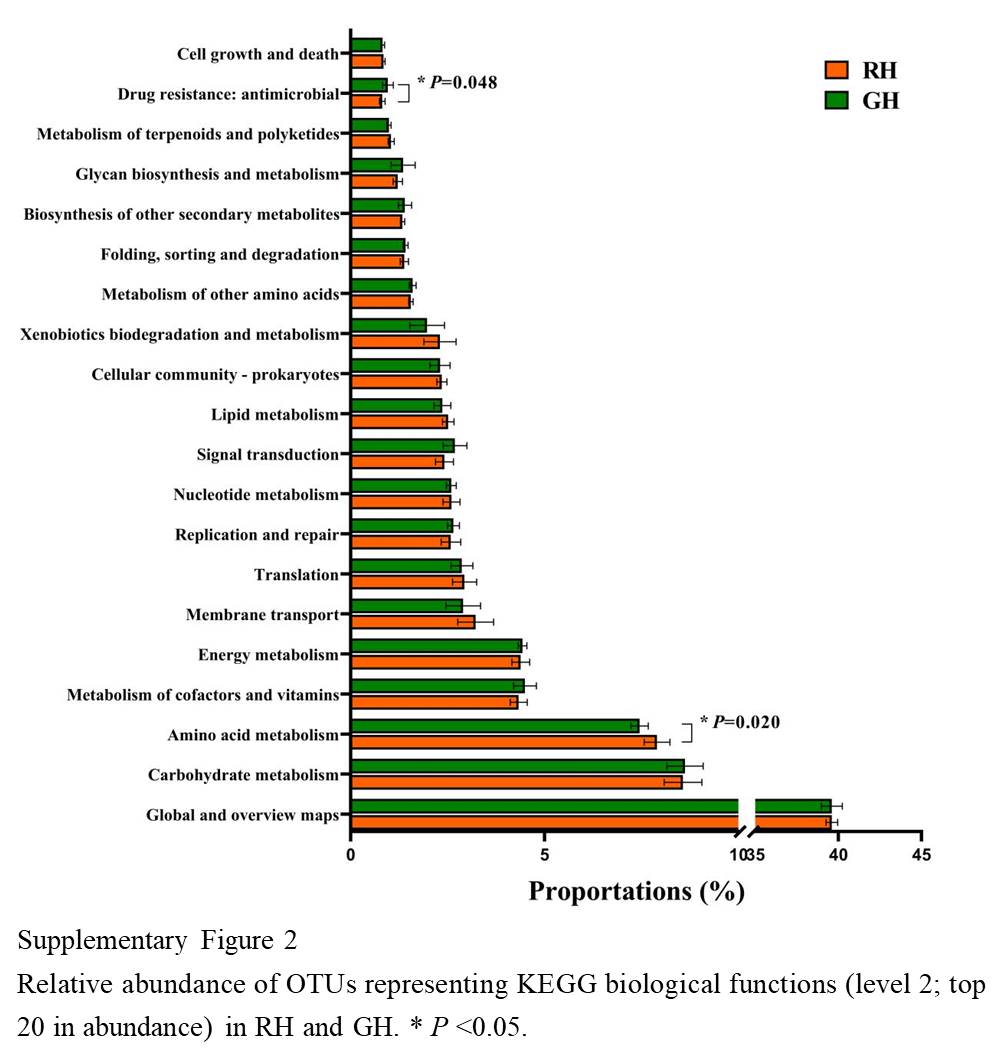

Supplement: Supplementary file 4 [file Image_2.jpg]
